# Bacteremia caused by accidental injection of *Bacillus licheniformis* microbiota modulator through the central venous catheter: A case report

**DOI:** 10.1097/MD.0000000000028719

**Published:** 2022-01-28

**Authors:** Chuan Zhong, Fen Wang, Haining Zhou, Jiarui Liu, Jiewei Hu, Yongjun Chen

**Affiliations:** a Department of Thoracic Surgery, Suining Central Hospital, Suining, Sichuan, China; b Clinical Laboratory, Suining Central Hospital, Suining, Sichuan, China; c Department of Gastroenterology, Suining Central Hospital, Suining, Sichuan, China.

**Keywords:** *Bacillus licheniformis*, bacteremia, central venous catheter

## Abstract

**Rationale:**

*Bacillus licheniformis* (*B licheniformis*) is a commonly used microbiota modulator. However, infections are rarely observed in immunocompetent hosts.

**Patient concerns:**

A 67-year-old woman who underwent esophagectomy experienced accidental injection of *B licheniformis* and presented with chills followed by hyperpyrexia.

**Diagnosis:**

The initial diagnosis was *B licheniformis* bacteremia.

**Intervention:**

Based on our experience, the patient first received levofloxacin and ornidazole. The application of levofloxacin was retained based on the antibiogram results. After discharge, the antibiotics were changed to vancomycin and levofloxacin, based on sensitivity tests, until two consecutive blood cultures were negative.

**Outcomes:**

The patient recovered without any severe complications.

**Lessons:**

This is a rare report of the successful treatment of *B licheniformis* bacteremia caused by improper drug administration, which will provide a reference for the treatment of *B licheniformis* bacteremia.

## 1 Introduction

Esophageal cancer is one of the most malignant cancers, with high morbidity and mortality rates.^[1]^ It is commonly classified into esophageal squamous cell carcinoma (ESCC) and esophageal adenocarcinoma based on histological characteristics, with ESCC being the most prevalent subtype in China.^[2]^ Although great progress has been made in the development of novel treatments for cancer, surgical resection remains the primary treatment for resectable ESCC.^[3]^ Pulmonary diseases, such as pneumonia, are a common complication after esophagectomy. Malnutrition, alveolar collapse, pulmonary edema, impaired defence systems, and poor ventilation are thought to contribute to the development of pulmonary infections.^[4]^

As a commonly used microbiota modulator, *Bacillus licheniformis* (*B licheniformis*) is demonstrated to attenuate dextran sulphate sodium (DSS)-induced colitis in mice and normalize ileum microbiota in infected chicken.^[5,6]^ Therefore, it is clinically used to maintain the balance of the intestinal microbiota. However, *B licheniformis* infection after esophagectomy is rarely observed. This report presents the successful treatment of an accidental infection of *B licheniformis* caused by improper drug administration, with a large dose of bacteria (∼250 million live bacteria) injected into the blood.

## 2 Case presentation

A 67-year-old woman was admitted to our hospital on March 6, 2017, complaining of dysphagia for three months. Esophagogastroscopy revealed an ulcerative lesion located 25 cm from the incisors, and a biopsy revealed squamous cell carcinoma. On enhanced thoracic and abdominal CT scans, the tumor was classified as clinical T3N0M0 (cT3N0M0, stage II). The patient underwent combined thoracoscopic–laparoscopic esophagectomy (left neck anastomosis) with thoracoabdominal two-field lymphadenectomy 3 days after admission. Deep venous catheterization was performed through the right internal jugular vein to facilitate venous transfusion before surgery and nasogastric and nasoduodenal tubes were placed in the proper position after anastomosis. Conventional therapies, including antacids, nutritional support, and maintenance of water and electrolyte balance, have been carried out.

**Figure 1:**
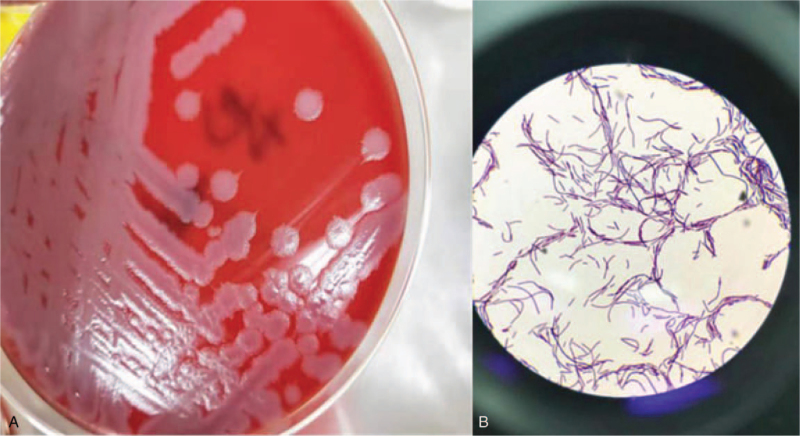
(A) Mucilaginous colonies of *Bacillus licheniformis* growing in Columbia agar. (B) Gram-positive bacilli detected under the microscope with Gram staining.

Three days after the surgery, the patient began to cough with abundant yellow sputum. CT suggested pneumonia in the left lower lobe, and semi-synthetic penicillin was administered. Considering that long-term application of antibiotics could lead to intestinal flora disorders, Zhengchangsheng (*B licheniformis*), a probiotic agent originally developed in China,^[7]^ was administered to maintain the balance of intestinal microbiota. One Zhengchangsheng capsule (*B licheniformis* 250 mg, including ∼250 million live bacteria) was diluted with pre-boiled warm water and inadvertently left by an overworked nurse in the patient's room. The medication should be injected through the nasogastric tube. However, the patient's daughter, without authorization or inquiry, mistakenly injected the medication into the blood through the central venous catheter at 1:30 pm on March 22, 2017. The patient's daughter was unaware of what she had done until the medication was injected. Soon after, the patient presented with chills followed by hyperpyrexia. The first diagnosis was bacteremia. Immediate actions were taken, including removal of the central venous catheter, blood analyses, C-reactive protein (CRP) and procalcitonin (PCT) measurements, blood culture, moderate acceleration fluid infusion, and antipyretic drug administration. The patient was transferred to the intensive care unit (ICU). A multidisciplinary team comprising thoracic surgeons, intensivists, pharmacologists, microbiologists, nephrologists, and gastroenterologists was formed. They reached a consensus to closely monitor vital signs, infection indicators, and hepatorenal function for the early detection of multiple organ dysfunction and to perform hemodialysis when necessary. Levofloxacin and ornidazole were administered according to previous reports.^[8]^ Besides, according to Clinical and Laboratory Standards Institute guidelines, two blood specimens, one from each side of the patient, were collected and cultured at 35°C for 48 hours using the bioMérieux automatic blood culture instrument (BacT/ALERT 3D), and the bacteria were detected. The bacterium was isolated, cultured in accordance with the National Clinical Examination Operating Procedures of China (4th edition), and transferred to a blood culture dish (Fig. 1A). Gram staining revealed the presence of gram-positive bacilli (Fig. 1B). The strain was sent to the Microbiology Laboratory of Sichuan Provincial People's Hospital for identification. The strain was identified as *B licheniformis* using an automated microbial identification system (VITEK MS, France). The antibiogram showed that the strain was sensitive to levofloxacin and vancomycin; moderately sensitive to amikacin and chloramphenicol; and resistant to ceftazidime and penicillin. Therefore, levofloxacin was retained. No serious complications were detected, and the patient was transferred to the ordinary ward 3 days later. After antibiotic therapy for nearly 1 month, *B licheniformis* was still detected in the blood cultures; therefore, antibiotics were changed to vancomycin and levofloxacin based on sensitivity tests. Simultaneously, ultrasound cardiography revealed no cardiac vegetation, suggesting no infective endocarditis. After two consecutive negative blood cultures, antibiotics were discontinued. The patient was discharged 59 days after the surgery. After 1 year of follow-up, the infection did not recur.

## 3 Discussion

Infection with *B licheniformis* is mainly reported in immunocompromised patients and is rarely observed in immunocompetent hosts.^[8]^ The Infection in immunocompetent individuals is mainly correlated with medical manipulations such as indwelling central venous catheters and non-sterile cotton wool,^[9,10]^ and external injury may also lead to *B licheniformis* infection. Ameur et al reported the case of a 50-year-old female who suffered from *B licheniformis* cutaneous infection due to a wicker splinter.^[11]^ Yuste et al reported a 5-year-old female with a penetrating injury on her left foot caused by plant thorns, which resulted in infection with *B licheniformis*.^[8]^ Since *B licheniformis* is an aerobic, gram-positive bacterium, it is sensitive to antibiotics such as cefepime, carbapenems, aminoglycosides, and vancomycin, which can be determined based on hemoculture, and barely any patient will die after antibiotic treatment.^[12]^

Moreover, there is usually a history of direct inoculation in immunocompetent individuals infected with *B licheniformis*, such as in central venous catheters and prosthetic valves.^[9,13]^ Lépine et al reported a suspected central catheter infection of *B licheniformis* because the patient quickly recovered after removal of the catheter.^[14]^ The glutamate polymer generated by *B licheniformis* can form a biofilm around the catheter to facilitate its invasion.^[15]^ To the best of our knowledge, this is the first report of a large dose of *B licheniformis* infection in patients after esophagectomy. The infection was managed quickly and efficiently, as the case history could help us quickly define the pathogen. Blood cultures revealed gram-positive bacilli, and the strain was confirmed. Therefore, treatment based on sensitivity tests successfully controlled bacteremia with no subsequent severe complications. The patient was satisfied with immediate intervention.

## 4 Conclusions

The occurrence of such accidents indicates that management needs improvement, and health education is necessary. Nevertheless, our case provides a reference for the treatment of *B licheniformis* bacteremia.

## Acknowledgments

The authors thank Ramón Rami-Porta, MD, PhD (Department of Thoracic Surgery, Hospital Universitari Mútua Terrassa, University of Barcelona) for editing this manuscript.

## Author contributions

**Conceptualization:** Chuan Zhong, Yongjun Chen.

**Supervision:** Yongjun Chen.

**Writing – original draft:** Chuan Zhong, Fen Wang, Haining Zhou.

**Writing – review & editing:** Jiarui Liu, Jiewei Hu.
